# Transcutaneous Electrical Acupoint Stimulation vs Metoclopramide for Moderate to Severe Postoperative Nausea and Vomiting: A Randomized Clinical Trial

**DOI:** 10.1001/jamasurg.2025.6394

**Published:** 2026-01-28

**Authors:** Dong-yu Zheng, Peng Ding, Ming Gong, Hong-wei Zhu, Hui-jing Shi, Guang-li Ren, Ling-yan Jin, Yong-qiang Wang, Hong-bin Yuan, Yong-hua Li

**Affiliations:** 1 Department of Anesthesiology, Second Affiliated Hospital of Naval Medical University (Shanghai Changzheng Hospital), Shanghai, China; 2 Department of Anesthesiology, The 983rd Hospital of the Chinese People’s Liberation Army Joint Logistics Support Force, Tianjin, China; 3 Department of Anesthesiology, Fifth People’s Hospital of Shanghai Fudan University, Shanghai, China; 4 Department of Anesthesiology, Shuguang Hospital Affiliated to Shanghai University of Traditional Chinese Medicine, Shanghai, China

## Abstract

**Importance:**

Moderate to severe postoperative nausea and vomiting (PONV) significantly impedes recovery and is inadequately managed by current pharmacotherapy.

**Objective:**

To evaluate the clinical efficacy of a novel wearable transcutaneous electrical acupoint stimulation (TEAS) device targeting the PC6 (Neiguan) acupoint in managing moderate to severe PONV.

**Design, Setting, and Participants:**

This randomized, double-dummy, patient-blinded and observer-blinded, parallel-group, and active-controlled clinical trial was conducted at 4 hospitals in Shanghai and Tianjin, China, from May 2024 through May 2025. Eligible participants were female patients aged 25 to 55 years scheduled for thyroidectomy or anterior cervical surgery undergoing general anesthesia who developed moderate to severe PONV (numerical rating score [NRS] ≥4). These data were analyzed during June 2025.

**Interventions:**

Participants were randomized 1:1 to receive either active TEAS via the EmeTerm wristband applied for 2 hours with a saline placebo injection or an identical inactive TEAS device (model stimulator) with intravenous metoclopramide, 10 mg. Participants not achieving symptom relief (NRS ≥4) at 2 hours underwent rerandomization to cross-intervention.

**Main outcome and measure:**

The primary outcome was the 2-hour PONV remission rate following initial intervention defined as a significant relief in symptoms (NRS ≤3).

**Results:**

A total of 310 patients were screened for eligibility and 232 (74.8%) were randomized into either TEAS group (n = 116) or control group (n = 116). All participants completed the trial and a per-protocol analysis was conducted. Among 232 total patients, mean (SD) age was 43.5 (5.1) years and 100% were female. The TEAS group demonstrated a significantly higher 2-hour remission rate (77.6%; 95% CI, 69.2%-84.2% vs 55.2%; 95% CI, 46.1%-63.9%; *P* < .001) and a lower 24-hour relapse rate (12.2%; 95% CI, 7.0%-20.6% vs 56.3%; 95% CI, 44.1%-67.7%; *P* < .001) compared with controls. No adverse events were reported.

**Conclusion and relevance:**

In this study, wearable TEAS significantly outperformed metoclopramide for moderate to severe PONV, representing an effective nonpharmacological alternative that enhances postanesthesia recovery.

**Trial Registration:**

Chinese Clinical Trial Registry Identifier: ChiCTR2400084329

## Key Points

**Question:**

Is wearable transcutaneous electrical acupoint stimulation effective in managing moderate to severe postoperative nausea and vomiting?

**Findings:**

In this multicenter randomized clinical trial involving 232 female patients who underwent thyroidectomy or anterior cervical surgery under general anesthesia, wearable transcutaneous electrical acupoint stimulation at the PC6 acupoint demonstrated significantly greater efficacy than metoclopramide in managing moderate to severe postoperative nausea and vomiting.

**Meaning:**

The patient-controlled wearable device represents an effective nonpharmacological alternative to enhance postanesthesia recovery.

## Teaser

This study evaluates the clinical efficacy of a novel wearable transcutaneous electrical acupoint stimulation device targeting the PC6 acupoint in managing moderate to severe postoperative nausea and vomiting.

## Introduction

Postoperative nausea and vomiting (PONV) remains a clinically significant challenge, frequently persisting despite conventional antiemetic regimens due to their suboptimal efficacy and associated adverse effects. These shortcomings have heightened interest in nonpharmacological interventions, including transcutaneous electrical acupoint stimulation (TEAS) at the PC6 (Neiguan) point, has gained increasing validation.^1^ Previous work from our group confirmed that a wearable TEAS device (EmeTerm) effectively delivers PC6 stimulation and reduces PONV incidence after hysteroscopic surgery—a prophylactic effect substantiated in later investigations.^2^

Nevertheless, existing studies have primarily evaluated TEAS as a preoperative prophylactic measure. However, in clinical practice, many patients express a preference for receiving treatment after PONV onset rather than as preemptive prophylaxis. To address this preference and bridge a key evidence gap, this study introduces a therapeutic paradigm, enrolling patients who developed moderate to severe PONV following general anesthesia in real-world settings. Through a multicenter randomized clinical trial, we rigorously assessed the efficacy of wearable TEAS as an on-demand therapy for active PONV.

Establishing TEAS as a validated nonpharmacological treatment for breakthrough PONV carries important clinical implications. Its integration into postoperative care may not only improve recovery quality and patient satisfaction through more responsive symptom control but also help reduce reliance on pharmacologic agents and associated health care costs.

## Methods

### Study Design

This patient-blinded and observer-blinded, parallel-group, active-controlled randomized trial was conducted at 4 hospitals in Shanghai and Tianjin, China, from May 2024 to May 2025. The protocol complied Consolidated Standards of Reporting Trials (CONSORT) reporting guidelines.^3^ Ethics approval was obtained from the Ethics Committee of the Second Affiliated Hospital of Naval Medical University (2023SL074) before participant recruitment. The trial was registered at the Chinese Clinical Trial Registry (ChiCTR2400084329). An independent data monitoring committee oversaw trial conduct and safety. All participants provided written informed consent.

### Study Population

Eligible participants were female patients aged 25 to 55 years with an American Society of Anesthesiologists (ASA) physical status of I or II and scheduled for elective thyroidectomy (for benign nodules or low-risk differentiated thyroid microcarcinoma) or anterior cervical discectomy and fusion (anterior cervical discectomy and fusion) for single-level or 2-level cervical spondylotic radiculopathy or myelopathy. The key inclusion criterion was the development of moderate to severe PONV, defined as a numerical rating scale (NRS) score of 4 or higher following surgery under general anesthesia.

Patients were excluded if they had severe hepatic or kidney dysfunction, contraindications to metoclopramide use, perioperative unstable vital signs, or if they declined to consent to acupoint electrical stimulation therapy.

### Randomization and Masking

Permuted block randomization was performed using variable block sizes from 4 to 10. Random numbers were generated using SAS version 9.2 (SAS Institute). Sequentially numbered opaque envelopes were prepared by a research nurse and opened after enrollment. Participants and data collectors were blinded to group allocation. Personnel administering TEAS or pharmacological interventions were unmasked but excluded from outcome assessment and had no contact with participants during data collection.

### Interventions

No premedication was administered to any participant. Upon entering the operating room, standard monitoring (electrocardiography, noninvasive blood pressure, and pulse oximetry) was established for all participants. General anesthesia was inducted with intravenous dexamethasone, 5 mg, sufentanil 0.4 μg.kg^−1^, propofol 2 mg.kg^−1^, and cisatracurium, 0.15 mg.kg^−1^, followed by orotracheal intubation. Anesthesia was maintained using target-controlled infusion of propofol and continuous remifentanil infusion. Intravenous dolasetron, 12.5 mg, was administered during surgical closure.

A blinded researcher recorded PONV severity using an 11-point NRS (0 = no symptoms; 10 = worst imaginable). Patients with moderate to severe PONV (NRS ≥4) were randomized to either the TEAS group or the control group.

Participants in TEAS group received active stimulation via the EmeTerm wristband (model YF-ZTY-E1; WAT Medical Enterprise)—a reusable and rechargeable device—with electrodes positioned at the PC6 acupoint, alongside intravenous saline placebo injection. The device delivers an adjustable output voltage ranging from 17.6 v to 43.2 v across 5 discrete intensity levels. Prior to each session, personalized stimulation parameters were established through a stepwise calibration process: starting at level 1, the intensity was gradually increased until the participant reported perceivable paresthesia within the median nerve distribution area, with the highest well-tolerated level set as the therapeutic intensity. In accordance with manufacturer instructions and institutional infection control policies, each device was assigned to a single patient throughout the study and disinfected with 70% alcohol wipes between applications.

Participants in the control group wore a visually identical but inactive sham device (model YF-ZTY-E1; WAT Medical Enterprise) and received intravenous metoclopramide, 10 mg. The sham device replicated all external features of the active stimulator—including dimensions, weight, and operational cues, such as light-emitting diode indicators and blinking patterns—to simulate an active state, but was electronically disabled and delivered no transcutaneous electrical current.

At the 2-hour follow-up, the remission rate of moderate to severe PONV (NRS ≤3) were evaluated. Patients achieving remission continued their assigned intervention for 24 hours to assess the relapse rate.

Nonresponders (NRS ≥4) underwent rescue crossover. The TEAS group nonresponders discontinued stimulation and were rerandomized to receive either metoclopramide, 10 mg, intravenously or equivalent volume saline placebo. The control group nonresponders were rerandomized to receive either intravenous metoclopramide, 10 mg, or equivalent-volume saline placebo.

The postoperative analgesic protocol in the surgical ward consisted of on-demand intravenous flurbiprofen, 50 mg, or parecoxib, 40 mg, for moderate to severe pain, which was provided upon patient request.

### Outcomes

#### Primary and Secondary Outcomes

The primary outcome was the 2-hour remission rate for moderate to severe PONV following intervention. Secondary outcomes included 24-hour relapse rate and the remission rate at 2 hours post–cross-intervention in initial nonresponders.

#### Safety Outcomes

Safety was evaluated in terms of the occurrence of adverse event, including device-related events (hand numbness, pain, and movement disorders; cutaneous anaphylaxis), and other events (severe gastrointestinal discomfort, abnormal changes in vital signs).

### Sample Size Calculation

Sample size was calculated for the primary outcome using PASS software version 2023 (NCSS). Based on our pilot study, metoclopramide achieved a 37.5% (6 of 16) 2-hour remission rate for moderate to severe PONV, while TEAS yielded a 60% (12 of 20) remission rate. This represented a 60% relative improvement over the metoclopramide baseline (37.5% × 1.6 = 60%). With a 2-sided α = .05 and 90% power, 104 participants were required. Accounting for 10% attrition due to protocol deviations/postoperative withdrawal, 232 patients (116 per group) were enrolled.

### Statistical Analysis

Data normality was tested by the Shapiro-Wilk test. The primary outcome was analyzed using χ^2^ test according to the principle of intention to treat. Binary and categorical variables were analyzed using χ^2^ or Fisher exact tests, according to cell value expectation. Ordinal data were analyzed using the Mann-Whitney (intergroup) or Wilcoxon tests (matched pairs). A 2-sided *P* value less than .05 was considered statistically significant. All statistical analyses were conducted using the SPSS software version 25.0 (IBM).

## Results

### Baseline Characteristics

Between May 2024 and May 2025, a predetermined anesthetic protocol was applied to 1050 female patients undergoing thyroid or anterior cervical surgery. Of these, 310 patients (29.5%) developed moderate to severe PONV. After applying inclusion/exclusion criteria, 232 patients (74.8%) were enrolled and randomized into 2 study groups (Figure). All procedures were performed by senior attending surgeons using standardized open or minimally invasive techniques, with a mean (SD) surgical duration of 91.3 (25.6) minutes and no reported major intraoperative complications, such as significant hemorrhage, recurrent laryngeal nerve injury, or esophageal injury. Baseline demographic and clinical characteristics, including ASA physical status, body mass index, and Apfel risk score, are listed in Table 1.

**Figure.**
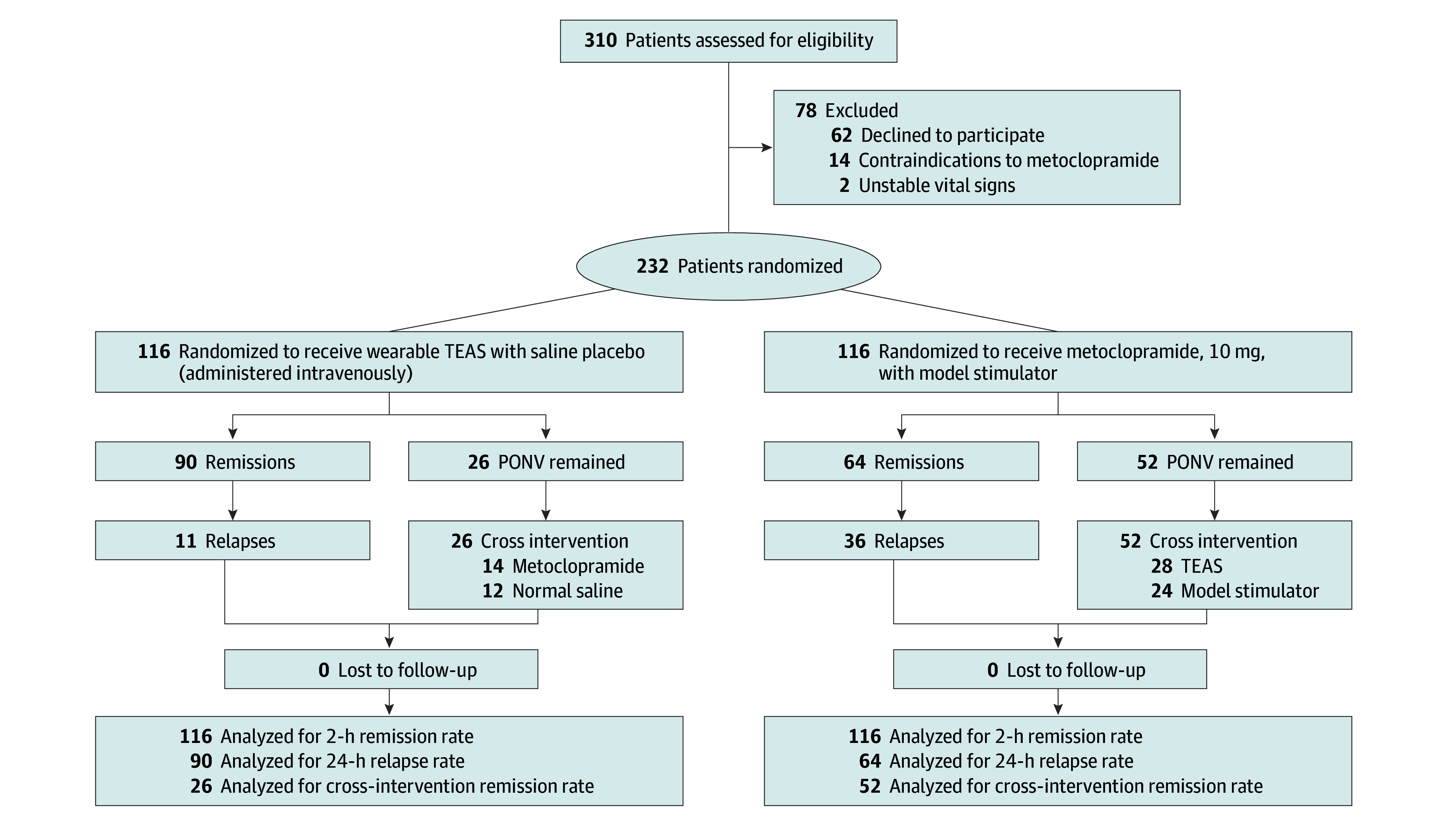
Flow Diagram PONV indicates postoperative nausea and vomiting; TEAS, transcutaneous electrical acupoint stimulation.

**Table 1.** Patient Characteristics and Surgical Data

Variable	No. (%)	
	TEAS (n = 116)	Control (n = 116)
Age, y, mean (SD)	43.3 (5.4)	42.6 (4.8)
Female sex	116 (100)	116 (100)
BMI,^a^ mean (SD)	20.3 (2.1)	20.1 (1.6)
ASA physical status		
I	70 (60.3)	65 (56.0)
II	46 (39.7)	51 (44.0)
Risk factors		
Nonsmoker	105 (90.5)	100 (86.2)
History of PONV/motion sickness	20 (17.2)	16 (13.8)
Postoperative opioids	0 (0)	0 (0)
Apfel scores		
2	107 (92.2)	106 (91.4)
3	18 (15.5)	10 (8.6)
4	0 (0)	0 (0)

Abbreviations: ASA, American Society of Anesthesiologists; BMI, body mass index; PONV, postoperative nausea and vomiting; TEAS, transcutaneous electrical acupoint stimulation.

^a^ Calculated as weight in kilograms divided by height in meters squared.

### Primary Outcome

TEAS therapy demonstrated superior efficacy in relieving moderate to severe PONV compared with controls, with a remission rate of 77.6% (95% CI, 69.2%-84.2%) vs 55.2% (95% CI, 46.1%-63.9%; *P* < .001). This translated to a 50% reduction in PONV risk with TEAS (risk ratio, 0.50; 95% CI, 0.34-0.73). The attributable risk difference was 22% (95% CI, 11.0%-35.0%), yielding a number needed to treat of 4.46 (95% CI, 2.85-9.42), indicating that 1 additional case of significant relief is achieved per 5 patients treated with TEAS (Table 2).

**Table 2.** 2-Hour Remission Rate and Numerical Rating Scale (NRS) of Postoperative Nausea and Vomiting in Patients Receiving Initial Intervention

Variable	TEAS (n = 116)	Control (n = 116)	*P* value
No. of remissions	90	64	NA
Remission rate, % (95% CI)	77.6 (69.2-84.2)	55.2 (46.1-63.9)	<.001
RR	0.50 (0.34-0.73)		NA
ARD	0.22 (0.11-0.35)		NA
NNT	4.46 (2.85-9.42)		NA
NRS,^a^ median (IQR)	7 (5-10)	7 (5-9.75)	.79
NRS,^b^ median (IQR)	2 (1-3)	3 (2-6)	<.001
*P* value of matched-pairs test^c^	<.001	<.001	NA

Abbreviations: ARD, attributable risk difference; NA, not applicable; NNT, number needed to treat; RR, relative risk; TEAS, transcutaneous electrical acupoint stimulation.

^a^ NRS preintervention.

^b^ NRS postintervention.

^c^ Differences of NRS between preintervention and postintervention were analyzed using the Wilcoxon signed rank test.

The observed changes in NRS further substantiated the above results: baseline NRS showed no significant difference between groups; NRS decreased in both groups after initial intervention, with significant intergroup differences (Table 2).

### Secondary Outcomes

Among patients achieving symptom relief through initial intervention, the 24-hour relapse rate was significantly lower in the TEAS group than controls (12.2% vs 56.3%; *P* < .001) despite comparable worst NRS scores (Table 3).

**Table 3.** 24-Hour Relapse Rate of Postoperative Nausea and Vomiting in Patients Achieving Remission After Initial Treatment

Variable	TEAS (n = 90)	Control (n = 64)	*P *value
No. of relapses	11	36	NA
Relapse rate, % (95% CI)	12.2 (7.0-20.6)	56.3 (44.1-67.7)	<.001
RR	0.22 (0.12-0.38)		NA
ARD	0.44 (0.31-0.60)		NA
NNT	2.27 (1.67-3.27)		NA
Worst NRS, median (IQR)	5 (4-5)	5 (4-5)	.71

Abbreviations: ARD, attributable risk difference; NA, not applicable; NNT, number needed to treat; NRS, numerical rating scale; RR, relative risk; TEAS, transcutaneous electrical acupoint stimulation.

Patients with refractory PONV demonstrated robust therapeutic responses following a crossover trial: 50% of TEAS–assigned patients achieved moderate to severe symptom remission 2 hours postmetoclopramide vs 0% with saline (*P* = .006). Correspondingly, 75% of control-assigned patients with refractory PONV remitted after active TEAS vs 8.3% with sham stimulation (*P* < .001; Table 4).

**Table 4.** Remission Rate of Postoperative Nausea and Vomiting in Patients Receiving Cross-Intervention

TEAS group	Metoclopramide (n = 14)	Normal saline (n = 12)	*P* value
No. of remissions	7	0	NA
Remission rate, % (95% CI)	50 (26.8-73.2)	0 (0-24.2)	.006
**Control group**	**TEAS wristband (n = 28)**	**Model stimulator (n = 24)**	
No. of remissions	21	2	NA
Remission rate, % (95% CI)	75 (56.6-87.3)	8.3 (1.5-25.9)	<.001

Abbreviations: NA, not applicable; TEAS, transcutaneous electrical acupoint stimulation.

### Safety Outcomes

A total of 144 patients received TEAS via the EmeTerm device (116 during initial intervention and 28 during crossover intervention). No device-related adverse events were observed. Furthermore, no other significant adverse events were reported in either study group.

## Discussion

This randomized clinical trial provides pioneering evidence that wearable TEAS wristband constitutes an effective therapeutic intervention for moderate to severe PONV following general anesthesia. Our data demonstrated superior efficacy of TEAS over conventional metoclopramide therapy, evidenced by a 22.4% absolute increase in 2-hour symptom resolution (77.6% vs 55.2%; *P* < .001) and a 44.1% reduction in 24-hour relapse rate by (12.2% vs 56.3%; *P* < .001). These robust effects position TEAS as a clinically valuable antiemetic modality.

The persistent clinical challenge of PONV management stems from its complex multifactorial pathogenesis. Current multimodal regimens remain suboptimal due to concurrent neurotransmitter dysregulation (particularly in dopaminergic and serotonergic pathways), impaired gastrointestinal motility, and opioid-related adverse effects.^4^ Our study augments the mounting evidence supporting acupoint stimulation-based interventions with proposed mechanisms encompassing: (1) μ-opioid receptor-mediated β-endorphin release,^5^ (2) dual regulation of adrenergic signaling and serotonin type 3 receptor neurotransmission,^6^ (3) vagal nerve stimulation–enhanced gastric motility,^7^ and (4) opioid-sparing effects through endogenous analgesia potentiation.^8^ This poly-pharmacological profile may explain its superior efficacy against refractory cases.

Methodologically, we selectively enrolled female patients undergoing thyroidectomy or anterior cervical spine surgery to optimize cohort homogeneity. This strategic design leveraged 6 key advantages: intraoperative neck hyperextension predisposing to PONV, standardized brief anesthesia duration, restricted intraoperative fluid administration, clean surgical wounds eliminating antibiotic confounders, minimal surgical trauma, and deliberate sampling of high-risk patients (Apfel score ≥2 comprised over 85% of the cohort). By maintaining conventional anesthesia protocols while achieving high patient compliance, this approach maximized ecological validity without compromising methodological rigor.

Therapeutic interventions followed the Fourth PONV Consensus Guidelines, with all participants receiving first-line combination prophylaxis (intravenous dexamethasone, 5 mg, with intravenous dolasetron, 12.5 mg).^9^ For breakthrough PONV, metoclopramide, 10 mg, was selected as active comparator based on its dual dopamine receptor/serotonin type 3 receptor pharmacology, established central antiemetic action via chemoreceptor trigger-zone modulation, grade A1 evidence status, and superior pharmacoeconomic profile compared with newer agents.^10^ This design addressed the well-documented plateau effect of serotonin antagonist dose escalation.^11^

Our crossover analysis revealed a critical therapeutic synergy between TEAS and conventional antiemetics. Among metoclopramide nonresponders, 75% (21 of 28) achieved remission with TEAS rescue, while 50% (7 of 14) of TEAS nonresponders subsequently responded to metoclopramide. This bidirectional efficacy positions TEAS as a versatile component of stepwise PONV management, effectively functioning as both first-line and rescue therapy. Our findings mark a paradigm shift by validating TEAS for active PONV treatment, thereby addressing a significant evidence gap and establishing a novel nonpharmacological strategy for moderate to severe cases.

The wearable TEAS wristband demonstrated exemplary safety with 0 device-related adverse events and 100% treatment completion. This favorable safety profile, coupled with its noninvasive nature and high patient acceptance supports its feasibility in busy perioperative settings.

### Limitations

We acknowledge several limitations. First, the generalizability of our findings is constrained by the gender-restricted sampling strategy for the reason noted above. Future multicenter trials should adopt gender-inclusive enrollment to enhance external validity. Second, there was an inherent risk of partial unblinding because the perceptible sensation of electrical stimulation in the active TEAS group could have revealed the assignment. Despite the use of identical-appearing sham devices, we cannot exclude the possibility that some participants, particularly those familiar with electrical stimulation, may have deduced their group allocation. Lastly, the predominantly mild postoperative pain associated with the included surgical procedures (anterior cervical and thyroid) limited the need for postoperative opioid analgesia. Consequently, the findings may not be generalizable to patient populations experiencing more severe postoperative pain.

Three priority research directions emerge: (1) evaluate the comparative effectiveness of TEAS-integrated multimodal therapy vs conventional first-line antiemetic regimens in larger cohorts, (2) investigate potential synergistic interactions between neuromodulatory acupoint stimulation and pharmacotherapeutic agents, and (3) assess the therapeutic efficacy of TEAS application in patient cohorts requiring sustained postoperative analgesia.

## Conclusions

This multicenter randomized clinical trial provides robust evidence that patient-administered, wearable TEAS represents an effective therapeutic alternative to pharmacologic rescue therapy for established moderate to severe PONV. Our findings demonstrate that this nonpharmacological approach not only yields superior and sustained symptom resolution compared with metoclopramide but also offers a favorable safety profile. By validating a practical, patient-centric model for on-demand PONV control, this study supports a paradigm shift toward nonpharmacological strategies in perioperative recovery, with the potential to enhance patient autonomy, reduce medication-related adverse effects, and improve the quality of postanesthesia care.

## Notes

Supplement 1. Study protocol

Supplement 2. Statistical analysis plan

Supplement 3. Data sharing statement
