# Supplementary material for: Transcutaneous Electrical Acupoint Stimulation vs Metoclopramide for Moderate to Severe Postoperative Nausea and Vomiting: A Randomized Clinical Trial
Source: JAMA Surg. 2026 Jan 28;161(3):268–73. doi: 10.1001/jamasurg.2025.6394 (PMC12853276; doi:10.1001/jamasurg.2025.6394)
Supplement: Supplement 1. — Study protocol [file jamasurg-e256394-s001.pdf]

STUDY PROTOCOL

**Title**	Efficacy of wearable transcutaneous electrical acupoint stimulation bracelet on moderate-to-severe postoperative nausea and vomiting in patients after general anesthesia: a multicenter randomized controlled trial
**Version**	2.0
**Date**	Dec 25, 2023
**Leading Center**	Second Affiliated Hospital of Naval Medical University (Shanghai Changzheng Hospital), Shanghai, China.
**Chief Investigator**	Yong-hua Li
**Principal Investigator**	Peng Ding, Ling-yan Jin, Yong-qiang Wang
**Participating Center**	1. The 983rd Hospital of the Chinese People's Liberation Army Joint Logistics Support Force, Tianjin, China.<br>2. Fifth People's Hospital of Shanghai Fudan University, Shanghai, China.<br>3. Shuguang Hospital Affiliated to Shanghai University of Traditional Chinese Medicine, Shanghai, China
**Funding**	The second round of the Shanghai Shenkang Hospital Development Center's "Three Year Action Plan to Promote Clinical Skills and Clinical Innovation in Municipal Hospitals" research-oriented physician innovation and transformation ability training project (SHDC2023CRD024), Youth Talent Program of Zhengzhou Joint Logistic Support Center (No.202310).

Confidentiality statement

This document is intended for the confidential review, deliberation and / or participation of the research trial and its designated representatives. Without the prior written authorization of the researcher or his representative, it shall not be copied or distributed to unauthorized personnel for review.

1. Introduction

1.1 Background and rationale

Postoperative nausea and vomiting (PONV) is the most common complication following general anesthesia, accounting for 43% of all inpatients, with an incidence rate of 70%-80% among high-risk patients [1-2]. Severe cases can lead to wound dehiscence, incisional hernia, aspiration pneumonia, asphyxia, and even death [3]. Pharmaceutical therapy is currently the primary method, with commonly used drugs including 5-HT3 receptor antagonists, glucocorticoids, dopamine receptor antagonists, substance P antagonists, anticholinergics, and antihistamines [1]. The multitude of drug types indicates the lack of a specific drug against PONV. Pharmaceutical therapy has reached a bottleneck, and the inherent adverse reactions of these antiemetic drugs, such as headache, dizziness, arrhythmia, coupled with relatively high drug costs, limit their widespread use. Therefore, it is urgent to explore clinically effective non-pharmaceutical therapies [4-5], including acupuncture, acupressure and transcutaneous electrical acupoint stimulation (TEAS). However, these traditional methods have high personnel skill and equipment requirements. Our previous randomized controlled trial using a wearable bracelet device based on the principle of TEAS found that it reduced the incidence of PONV in patients undergoing hysteroscopic surgeries [6]. Although previous studies have suggested that the TEAS bracelet can prevent PONV, its effectiveness on PONV that has already occurred remains unknown. Thus, we aim to study the therapeutic effect of the TEAS bracelet on patients who have suffered moderate-to-severe PONV after general anesthesia in real-world settings.

1.2 Objectives

The objective of this study is to evaluate the therapeutic effect of the TEAS bracelet on moderate-to-severe PONV in patients after general anesthesia. Our hypothesis is that the wearable non-pharmaceutical device is superior to metoclopramide treatment.

1.3 Trial design

The trial is designed as a randomized, controlled, patient-blinded, multicenter, superiority trial with two parallel groups. The primary endpoint of the trial is symptom relief during two hours after moderate-to-severe PONV. Randomization will be performed using block randomization with a 1:1 allocation ratio.

2. Methods

2.1 Participants

Study setting

The trial will be conducted in Shanghai and Tianjin, China. A total of 232 participants will be recruited from four academic hospitals: 82 participants from the Second Affiliated Hospital of Naval Medical University in Shanghai, 50 participants from Shanghai Shuguang Hospital, 50 participants from Shanghai Fifth People's Hospital, and 50 participants from PLA No.983 Hospital in Tianjin.

Eligibility criteria

Female patients undergoing thyroidectomy or anterior cervical surgery under general anesthesia will be screened for participation.

Inclusion criteria

- Moderate to severe PONV (visual analogue score ≥4) within 24 hours after surgery
- Age 25-55 years
- American society of Anesthesiologists (ASA) physical status I-II

Exclusion criteria

- Severe hepatic or renal dysfunction;
- Declined to consent to acupoint electrical stimulation therapy;
- Contraindications to metoclopramide use;
- Perioperative unstable vital signs.

Informed consent

In this study, an anesthesiologist will approach patients who are eligible for participation. After explaining the general anesthesia procedure and obtaining consent, patients will be informed about the TEAS approach or medication to treat PONV. Patients will only be enrolled in the study if they provide consent for both the general anesthesia and the research. If a patient is unable to provide written consent, it will be obtained from their designated representative. Patients who refuse to

participate in the study will still receive the same quality of care as participants. Participants have the right to withdraw from the study at any time, in accordance with the Declaration of Helsinki (2013 version).

Additional consent provisions for collection and use of participant data and biological specimens

Not applicable. We will not collect any biological specimens from participants, and we are not currently considering the use of these data for ancillary studies.

Sample size

The sample size for this trial was calculated based on the primary outcome. We previously found that metoclopramide was routinely administered intravenously in patients suffering from moderate-to-severe PONV, and the effective response rate was 37.5% (6/16) after 2 hours of follow-up. We estimate that the effective rate will be increased to 60% by using the TEAS bracelet. Based on the primary outcome (37.5% vs. 60%), test power ($1-\beta=90\%$), and bilateral significance level ($\alpha=5\%$), a minimum of 104 samples are required for each group using PASS software. To account for a 10% dropout rate, we plan to recruit 232 participants (116 in each group) for this trial.

Recruitment

Each center participating in the trial has a dedicated anesthesiologist who is responsible for screening all women undergoing thyroid and anterior cervical surgery, particularly those with more than two Apfel's PONV risk factors. Participants are provided with detailed information about the potential complications of PONV, current mainstream treatments, and the potential benefits and risks of participating in the trial. The number of subjects enrolled in each center is determined based on the type and number of operations performed at the center, ensuring that the target sample size can be achieved across all four centers by the end of the enrollment period.

2.2 Interventions

Comparators

The Fourth Consensus Guidelines for the Management of PONV recommend the use of multimodal prophylaxis in patients with one or more risk factors [1]. In this study, both groups will be routinely treated with a combination of dexamethasone and dolasetron, which are the two most commonly used classes of drugs: glucocorticoids and serotonin agonists. For participants who have already developed PONV, repeated use or higher doses of these drugs often have little benefit. Therefore, we have chosen a new type of antiemetic, metoclopramide, as the comparative intervention in the control group. Metoclopramide is a dopamine 2 (D2) receptor antagonist and 5-hydroxytryptamine 4 (5-HT4) receptor agonist, and has a mild inhibitory effect on the 5-HT3 receptor. It acts on the dopamine receptor in the chemoreceptor trigger zone of the medulla to increase the threshold of the chemoreceptor trigger zone, and has a central antiemetic effect. It is a drug recommended in the guidelines (Evidence A1) and its price is relatively low, so we chose metoclopramide 10 mg as the comparative intervention in the control group.

Intervention description

All enrolled patients in this study will be routinely administered intravenously with dexamethasone 5 mg at the induction of general anesthesia, and dolasetron 12.5 mg at the end of surgery. Patients with moderate to severe PONV (VAS score ≥4) will be allocated into either the TEAS group or the control group.

In the TEAS group, patients with PONV (VAS score ≥4) will wear an EmeTerm bracelet and be injected with normal saline. They will be followed up 2 hours later to observe the response rate of PONV. If PONV is significantly relieved (VAS≤3), the intervention will be continued for 24 hours, and the recurrence rate of moderate and severe PONV within 24 hours will be observed and the highest score will be recorded.

In the control group, patients will wear a model bracelet (the light will flash normally, but there will be no electrical stimulation) and will be injected with metoclopramide 10 mg. Two hours later, the response rate of moderate to severe

PONV will be observed. If PONV was significantly relieved (VAS≤3), the recurrence rate of moderate and severe PONV within 24 hours will be observed and the highest score will be recorded.

In the TEAS group, patients who have no significant relief after the intervention of the electrostimulation bracelet (VAS score≥4) will stop using the bracelet and then be randomly divided into two groups. One group will be injected with metoclopramide 10 mg and the other with an equal-volume of normal saline. They will be followed up 2 hours later to observe the response rate of moderate and severe PONV.

In the control group, patients who have no significant relief after metoclopramide medication (VAS score≥4) will be randomly divided into two groups. One group will wear the electrical stimulation bracelet and the other will wear the model bracelet. They will be followed up 2 hours later to observe the response rate of moderate and severe PONV.

Criteria for discontinuing or modifying allocated interventions

If the vital signs of a participant are unstable after enrollment or if the participant does not cooperate with follow-up, the intervention will be suspended. Furthermore, any participant may withdraw from the trial at any time without providing a reason.

Strategies to improve adherence to interventions

The control group's intervention, which is a single intravenous injection of metoclopramide, has good compliance. The intervention in the TEAS group, which involves wearing a bracelet, typically does not affect the participant's activity and has a short duration of two hours. Therefore, adherence to the intervention is expected to be good. The investigators will ask the ward nurses to monitor whether the patients are wearing the bracelet properly during routine rounds.

Relevant concomitant care permitted or prohibited during the trial

Participants in both groups received conventional postoperative care. No additional antiemetics (5-HT3 receptor antagonists, glucocorticoids, dopamine receptor antagonists, substance P antagonists, anticholinergics, and antihistamines) will be

given. Ondansetron 4 mg will be administered intravenously as rescue medication.

Provisions for post-trial care

The intervention in this trial involved the normal use of a long-marketed product, while the control intervention was clinical routine medication. Participants are not expected to suffer harm from trial participation, and no special provisions for post-trial care are required.

Participant timeline

The overall schedule of enrolment, allocation, intervention, and follow-up is presented as a schematic diagram in Figure 1.

	STUDY PERIOD						
	Enrolment	Allocation	Post-allocation				Close-out
**TIMEPOINT**	$-t_1$	0	t_1	t_2	t_3	t_4	t_x
**ENROLMENT:**							
Eligibility screen	X						
Informed consent	X						
Allocation		X					
**INTERVENTIONS:**							
TEAS group			X				
Control group			X				
**CROSS INTERVENTIONS:**							
TEAS group					X		
Control group					X		
**ASSESSMENTS:**							
Response rate				X		X	X
Visual analogue score				X		X	X
Adverse events			X	X	X	X	X

Figure 1 Schematic diagram. Timepoint: $-t_1$, before anesthesia; 0, allocation; t_1: primary intervention (after PONV occurs and last for two hours); t_2: two hours after intervention; t_3, cross intervention (last for two hours); t_4: two hours after cross intervention.

2.3 Outcomes

The primary outcome of the trial is the response rate of moderate-to-severe postoperative PONV after 2 hours of intervention. This includes complete response, which is defined as the disappearance of all uncomfortable symptoms, and partial response, which is defined as the transition from vomiting to nausea or a significant reduction in the degree of nausea (VAS≤3). The secondary outcomes include the recurrence rate of moderate-to-severe PONV within 24 hours after intervention, as well as the response rate of moderate-to-severe PONV at 2 hours after cross-intervention in a population insensitive to the initial intervention.

2.4 Allocation

Sequence generation

Participants in the trial will be randomly assigned to either the control group or the TEAS group in a 1:1 ratio using sealed envelopes. Permuted block randomization will be performed using variable block sizes ranging from 4 to 10. The random numbers will be generated using SAS software to ensure the randomization process is unbiased and reliable.

Concealment mechanism

Allocation concealment will be ensured by using sealed opaque envelopes that will be kept by a research nurse. The allocation of participants to either the control group or the TEAS group remains unknown to both the participants.

Implementation

The statistical expert from the main research center (The Second Affiliated Hospital of Naval Medical University) will be responsible for generating the allocation sequence, preparing the envelopes, and sending them directly to the research nurses in each center. Participants will be enrolled by the anesthesiologist who administered the anesthesia, and will be assigned to the interventions by a specialized anesthesiologist who will be blinded to the group allocation.

2.5 Blinding

Who will be blinded

In this trial, both the patients and the follow-up researcher will be kept blind about the group allocation and interventions. A designated anesthesiologist will be responsible for postoperative follow-up, but will not interfere with clinical anesthesia and PONV treatment. After the end of the trial, the data will be summarized and sent to the full-time statistical personnel for analysis.

Procedure for unblinding if needed

Emergency unblinding is not involved in this trial because the safety of the intervention has been fully validated and does not affect routinely postoperative treatment and rehabilitation.

2.6 Data collection and management

Plans for assessment and collection of outcomes

Independent investigators at each center will be responsible for following up with the participants at 2 and 24 hours after the intervention. They will fill out case report files and print them out for storage. The case report files from each center will then be sent to the statistical expert of the leader unit for further analysis.

Plans to promote participant retention and complete follow-up

Follow-up in this trial will generally be completed within 24 to 48 hours after surgery, during which time participants are usually still hospitalized, so the investigator can easily conduct face-to-face follow-up. If a participant has any concerns or wishes to discontinue participation, he or she can contact his or her physician-in-charge or the study administrator at any time for assistance.

Data management

Case report files are filled out by an independent investigator and stored in a locked cabinet in each center, and these documents are not seen by study administrator, recruiters, allocation and intervention implementers. A research nurse enters the data into a computer and sends it directly to statistical expert.

Confidentiality

The case report files will be stored in a locked cabinet accessible only to the investigators. Personal information that may reveal the privacy of participants will not be recorded in the file.

Plans for collection, laboratory evaluation and storage of biological specimens for genetic or molecular analysis in this trial/future use

No biological specimens will be collected in this trial.

2.7 Statistical methods

Statistical methods for primary and secondary outcomes

The primary outcome will be analysed according to the intention-to-treat principle, and worst-case imputation will be used when there are missing data. Data normality is measured using the Shapiro-Wilk test. The intergroup differences are analysed using the Student's t-test or Mann-Whitney U test according to the data distribution. Binary and categorical variables are analysed using Fisher's exact tests. Ordinal data are analysed using the Wilcoxon signed-rank test. A two-sided P value less than 0.05 is considered statistically significant.

Interim analyses

No interim analyses are planned in the trial.

Methods for additional analyses

Participants who are not sensitive to the initial intervention will receive cross-intervention, and the additional outcome is the remission rate of PONV at 2 hours after cross-intervention, which will be analysed using Fisher's exact tests.

Methods in analysis to handle protocol non-adherence and any statistical methods to handle missing data

The primary outcome will be analysed according to the intention-to-treat principle, and worst-case imputation will be used when there are missing data. A per-protocol analysis will be performed to enhance the results.

Plans to give access to the full protocol, participant level-data and statistical code

This protocol is publicly available and the data sets generated/anal analysed during the current study are available from the corresponding authors on reasonable request.

3. Oversight and monitoring

Composition of the coordinating center and trial steering committee

The trial is led by the Department of Anesthesiology of the Second Affiliated Hospital of Naval Medical University and involve four participating centers. A senior professor from the leading unit and the administrators of the other three centers form a coordinating center that will meet online once a week to coordinate the progress of the trial, review the inclusion and exclusion criteria, and oversee the consent, recruitment, and follow-up process, without knowing the allocation, intervention, and outcomes of any specific participant.

Composition of the data monitoring committee, its role and reporting structure

The trial does not require a data monitoring committee because of the short duration and minimal known risks.

Adverse event reporting and harms

Possible adverse events in this trial include numbness in the palms and fingers, allergies to the electrodes or silicone strap, allergies to metoclopramide or related lethargy, irritability, and fatigue. These adverse events are usually mild and recover quickly after the intervention. Adverse events and harms will be recorded by the investigator and eventually included as categorical data in the safety outcome analysis.

Frequency and plans for auditing trial conduct

Audit is not planned in this trial.

Plans for communicating important protocol amendments to relevant parties

The trial will follow the latest version of the protocol. Any changes to the protocol or informed consent will be considered as amendments and version updates. These changes will be submitted for review to the Ethics Committee of Shanghai

Changzheng Hospital and the ethics committees of the other three centers.

Dissemination plans

The results of this trial will be communicated through conference presentation and publication of peer-reviewed research article.

References

[1] Gan T, Belani K, Bergese S, Chung F, Diemunsch P, Habib A, *et al*: Fourth consensus guidelines for the management of postoperative nausea and vomiting. *Anesthesia and analgesia* 2020, 131(2):411-448.

[2] Apfel C, Heidrich F, Jukar-Rao S, Jalota L, Hornuss C, Whelan R, *et al*: Evidence-based analysis of risk factors for postoperative nausea and vomiting. *Br J Anaesth* 2012, 109(5):742-753.

[3] Habib A, Chen Y, Taguchi A, Hu X, Gan T: Postoperative nausea and vomiting following inpatient surgeries in a teaching hospital: a retrospective database analysis. *Current medical research and opinion* 2006, 22(6):1093-1099.

[4] Chen J, Tu Q, Miao S, Zhou Z, Hu S: Transcutaneous electrical acupoint stimulation for preventing postoperative nausea and vomiting after general anesthesia: A meta-analysis of randomized controlled trials. *Int J Surg* 2020, 73:57-64.

[5] Stoicea N, Gan T, Joseph N, Uribe A, Pandya J, Dalal R, *et al*: Alternative therapies for the prevention of postoperative nausea and vomiting. *Frontiers in medicine* 2015, 2:87.

[6] Wang N, Ding P, Zheng DY, Pu J, Yang LY, Zhou YY, *et al*: Wearable transcutaneous electrical acupoint stimulation bracelet for prevention of postoperative nausea and vomiting in patients undergoing hysteroscopic surgery: a randomised controlled trial. *Br J Anaesth* 2022, 129(4):e85-e87.
