# Supplementary material for: Transcutaneous Electrical Acupoint Stimulation vs Metoclopramide for Moderate to Severe Postoperative Nausea and Vomiting: A Randomized Clinical Trial
Source: JAMA Surg. 2026 Jan 28;161(3):268–73. doi: 10.1001/jamasurg.2025.6394 (PMC12853276; doi:10.1001/jamasurg.2025.6394)
Supplement: Supplement 2. — Statistical analysis plan [file jamasurg-e256394-s002.pdf]

STATISTICAL ANALYSIS PLAN

Efficacy of wearable transcutaneous electrical acupoint stimulation bracelet on moderate-to-severe postoperative nausea and vomiting in patients after general anesthesia: a multicenter randomized controlled trial

Version: 1.0

Date: Dec 27, 2023

Protocol Version: 2.0

Protocol date: Dec 25, 2023

1. Introduction

Postoperative nausea and vomiting (PONV) is the most common complication following general anesthesia, accounting for 43% of all inpatients, with an incidence rate of 70%-80% among high-risk patients. Severe cases can lead to wound dehiscence, incisional hernia, aspiration pneumonia, asphyxia, and even death. Pharmaceutical therapy is currently the primary method, with commonly used drugs including 5-HT3 receptor antagonists, glucocorticoids, dopamine receptor antagonists, substance P antagonists, anticholinergics, and antihistamines. The multitude of drug types indicates the lack of a specific drug against PONV. Pharmaceutical therapy has reached a bottleneck, and the inherent adverse reactions of these antiemetic drugs, such as headache, dizziness, arrhythmia, coupled with relatively high drug costs, limit their widespread use. Therefore, it is urgent to explore clinically effective non-pharmaceutical therapies, including acupuncture, acupressure and transcutaneous electrical acupoint stimulation (TEAS). However, these traditional methods have high personnel skill and equipment requirements. Our previous randomized controlled trial using a wearable bracelet device based on the principle of TEAS found that it reduced the incidence of PONV in patients undergoing hysteroscopic surgeries. Although previous studies have suggested that the TEAS bracelet can prevent PONV, its effectiveness on PONV that has already occurred remains unknown.

2. Study aims and objectives

This trial aims to include female patients who have suffered from PONV after general anesthesia in real-world settings to investigate the therapeutic effect of the TEAS bracelet. The objective of this study is to evaluate the therapeutic effect of the TEAS bracelet on moderate-to-severe PONV in patients after general anesthesia.

3. General study design

The trial is designed as a double-dummy, randomized, active-controlled,

patient/obsever-blinded, multicenter, superiority trial with two parallel groups. The hypothesis is that the wearable non-pharmaceutical device is superior to metoclopramide treatment.

4. Interventions

Participants were randomized 1:1 to receive either active TEAS via the EmeTerm wristband applied for two hours plus saline placebo injection, or an identical inactive TEAS device (model stimulator) plus intravenous metoclopramide (10 mg). Participants not achieving symptom relief (NRS ≥4) at 2 hours underwent re-randomization to cross-intervention.

More details is provided in the trial protocol.

5. Changes of status

Full inclusion and exclusion criteria are described in the protocol.

Any participant who was randomised but became ineligible prior to the initiation of surgery will be classified as a post-randomisation exclusion. Such cases will be documented and reported, will not be included in the sample size calculation, and will be replaced through subsequent randomisations.

Participants who withdraw from further follow-up will be categorised as having declined continuation. Their subsequent data will be treated as missing, and they will not be replaced via over-recruitment.

6. Randomization, allocation, and blinding

Participants in the trial will be randomly assigned to either the control group or the TEAS group in a 1:1 ratio using sealed envelopes. Permuted block randomization will be performed using variable block sizes ranging from 4 to 10. The random numbers will be generated using SAS software to ensure the randomization process is unbiased and reliable.

In this trial, both the patients and the follow-up researcher will be kept blind about the group allocation and interventions. A designated anesthesiologist will be responsible for postoperative follow-up, but will not interfere with clinical anesthesia and PONV treatment. After the end of the trial, the data will be summarized and sent to the full-time statistical personnel for analysis.

7. Outcomes

The primary outcome of the trial is the response rate of moderate-to-severe postoperative PONV after 2 hours of intervention. This includes complete response, which is defined as the disappearance of all uncomfortable symptoms, and partial response, which is defined as the transition from vomiting to nausea or a significant reduction in the degree of nausea (VAS≤3).

The secondary outcomes include the recurrence rate of moderate-to-severe PONV within 24 hours after intervention, as well as the response rate of moderate-to-severe PONV at 2 hours after cross-intervention in a population insensitive to the initial intervention.

8. Schedule of data collection

The overall schedule of enrolment, allocation, intervention, and follow-up is presented as a schematic diagram in Figure 1.

	STUDY PERIOD						
	Enrolment	Allocation	Post-allocation				Close-out
TIMEPOINT	$-t_1$	0	t_1	t_2	t_3	t_4	t_x
ENROLMENT:							
Eligibility screen	X						
Informed consent	X						
Allocation		X					
INTERVENTIONS:							
TEAS group			X				
Control group			X				
CROSS INTERVENTIONS:							
TEAS group					X		
Control group					X		
ASSESSMENTS:							
Response rate				X		X	X
Visual analogue score				X		X	X
Adverse events			X	X	X	X	X

Figure 1 Schematic diagram. Timepoint: $-t_1$, before anesthesia; 0, allocation; t_1: primary intervention (after PONV occurs and last for two hours); t_2: two hours after intervention; t_3, cross intervention (last for two hours); t_4: two hours after cross intervention.

9. Adverse events

Possible adverse events in this trial include numbness in the palms and fingers, allergies to the electrodes or silicone strap, allergies to metoclopramide or related lethargy, irritability, and fatigue. These adverse events are usually mild and recover quickly after the intervention. Adverse events and harms will be recorded by the investigator and eventually included as categorical data in the safety outcome analysis.

10. Sample size and power calculation

The sample size for this trial was calculated based on the primary outcome. We previously found that metoclopramide was routinely administered intravenously in patients suffering from moderate-to-severe PONV, and the effective response rate

was 37.5% (6/16) after 2 hours of follow-up. We estimate that the effective rate will be increased to 60% by using the TEAS bracelet. Based on the primary outcome (37.5% vs. 60%), test power (1-β=90%), and bilateral significance level (α=5%), a minimum of 104 samples are required for each group using PASS software. To account for a 10% dropout rate, we plan to recruit 232 participants (116 in each group) for this trial.

11. Statistical methods

11.1 General methods

All analyses will follow the intention-to-treatment (ITT) principle. The final analysis will be conducted after all recruitment and follow-up are completed. The results of the trial will be reported in accordance with the CONSORT guidelines. Baseline data will be summarized using appropriate descriptive statistics according to the data distribution. Data normality will be tested by Shapiro-Wilk test. Treatment effects will be presented with 95% confidence intervals. All eligible participants will be included in the analysis, while post-randomization exclusions will be reported as such and not included in the analysis.

11.2 Interim analysis

No interim analysis are planned.

11.3 Primary outcome

The remission rate of initial intervention will be analysed using Chi-squire test or Fisher's exact tests according to cell value expectation. The NRS scores will be compared using Mann-Whitney test (inter-group) or Wilcoxon test (matched pairs). A two-sided *P* value less than 0.05 was considered statistically significant.

11.4 Secondary outcomes

The 24-hour relapse rate will be calculated in the population who received relief from initial intervention, and will be analysed using Chi-squire test or Fisher's exact tests according to cell value expectation.

Non-responders will receive a rescue crossover and re-randomization, the remission

rate of crossover intervention will be analysed using Chi-squire test or Fisher's exact tests according to cell value expectation.

11.5 Safety outcomes

Safety data will be presented for all participants who are randomized and receive intervention. The number of adverse events and serious events, as well as the proportion of participants with an event will be presented.

11.6 Missing data

The worst-case imputation will be used for missing outcome data,that is, if a participant in the TEAS group has no data due to dropout the follow-up, he will be recorded as no response to TEAS. On the contrary, control group participants with missing data were recorded as being positive to drug treatment.

11.7 Statistical software

All statistical analyses were conducted using the SPSS software (version 25.0, IBM Corporation, Armonk, NY, USA).
