# Supplementary material for: Transcutaneous Electrical Acupoint Stimulation vs Metoclopramide for Moderate to Severe Postoperative Nausea and Vomiting: A Randomized Clinical Trial
Source: JAMA Surg. 2026 Jan 28;161(3):268–73. doi: 10.1001/jamasurg.2025.6394 (PMC12853276; doi:10.1001/jamasurg.2025.6394)
Supplement: Supplement 3. — Data sharing statement [file jamasurg-e256394-s003.pdf]

Data Sharing Statement

Zheng. Transcutaneous electrical acupoint stimulation vs metoclopramide for moderate to severe postoperative nausea and vomiting. *JAMA Surg*. Published January 28, 2026. doi:10.1001/jamasurg.2025.6394

Data
Additional Information: This trial was registered at Chinese Clinical Trial Registry (ChiCTR2400084329), https://www.chictr.org.cn/showproj.html?proj=210862
Data available: Yes
Data types: Deidentified participant data
How to access data: liyonghua1207@smmu.edu.cn
When available: With publication

Supporting Documents
Document types: Informed consent form
How to access documents: liyonghua1207@smmu.edu.cn
When available: With publication

Additional Information
Who can access the data: researchers whose proposed use of the data has been approved
Types of analyses: for any purpose or for a specified purpose
Mechanisms of data availability: after approval of a proposal
